# Cyanosis Neonatorum

**Published:** 1859-01

**Authors:** A. G. Thomas

**Affiliations:** Atlanta, Ga.


### ARTICLE II.

*Cyanosis Neonatorum.* By A. G. THOMAS, A. M., M. D. Atlanta, Ga.

In a work entitled "Obstetrics—The Science and the Art." by Dr. Meigs, of Philadelphia, he has a very interesting article on Cyanosis, in which he introduces a method, original with him, to relieve the unfortunate neonatus from the usual fatal termination of this malady.

His treatment is very simple and has the sanction of anatomical reasonings—it is this: place the infant on its right side with the head very slightly elevated. This treatment has been successful in the hands of many other Physicians.

But in a case which has recently come before me, I have not been so fortunate, and hence I desire to bring it before the profession, for, whereas, the practice of Dr. Meigs has been so available in the majority of cases, I found it to fail in this case, but a good result was obtained by a very different course. The case is as follows:

On the night of the 17th of the present month, (Dec.) I was called to see a lady in a premature labor. I was informed that she was in her seventh month of gestation. After an easy labor she was delivered of a small child, weighing about three or four pounds, I thought when I received the child that it was dead, but on examination I found its heart pulsating freely but very rapidly. Knowing the danger of Cyanosis to children of premature birth, I immediately blew in its mouth and turned it on its right side and gave it to the nurse, when it cried faintly.

I then turned to the mother to take away the placenta, which I did very safely in a very short while. Having seen the mother cared for, I turned to the infant, and found it lying *right*, as I thought, but it was not breathing; its face was perfectly livid; it gasped at intervals of some minutes, and the heart scarcely beat. What to do, now that the great method failed, I knew not. I thought it would die in a short while, but knowing that the parents expected me to do something, I turned it on its *left side*, with no reason for it whatever, except to do something, for as I have already said, the plan of Dr. M. is sustained by reason. I let it remain so for a while, and I was very much astonished in a few minutes to hear the child cry out. I left it lying on its left side, and its breathing was established fully. In about an hour I left the house, when the child was breathing regularly and easily; in fact, doing well.

This child was certainly suffering from partial asphyxia, and every indication of cyanosis. I do not think it was from atelectasis, for the infant cried after I had inflated its lungs while lying on the right side.

Now the question is, why did not the placing of the child on the right side close the foramen ovale, and force the blood of the right auricle to pass beneath the tricuspid valves, and thereby through the right ventricle and pulmonary artery, so as to be ærated in the lungs?

I will leave this question with the profession, hoping soon to hear from some member of it, the true rationale of this anomalous case.

